# Hematuria after inguinal hernia repair in a child: a case report

**DOI:** 10.1186/s12887-024-04861-5

**Published:** 2024-06-07

**Authors:** Meng He, Jun He, Ning Li

**Affiliations:** 1 Department of Urology, Beijing Children’s Hospital, Capital Medical University, National Center for Children’s Health, Street No. 56, Beijing, 100045 China; 2 Foshan Women and Children Hospital, Foshan, China

**Keywords:** Inguinal hernia repair, Bladder injury, Child

## Abstract

Inguinal hernia repair is one of the most common surgical procedures in the pediatric population. While a rare complication, bladder injury can impose a significant burden on patients. This study outlined a case of bladder injury following selective inguinal hernia repair and summarized methods to prevent this complication, aiming to emphasize the importance of not underestimating interventions labeled as “routine surgery” in order to avoid avoidable harm to patients.

## Introduction

As is well documented, inguinal hernia is a prevalent condition in the pediatric population, with an incidence ranging between 1 and 5% [1] and reaching up to 30% in low-weight preterm infants [2]. Given this high prevalence, inguinal hernia repair remains one of the most common pediatric surgeries. The incidence of bladder injury during inguinal hernia repair has been estimated to range between 0.08% and 0.3% [3]. Although these complications are rare, they may lead to severe consequences and potentially result in lifelong disability [3–7]. This study documented a case of bladder injury in a child during selective inguinal hernia repair and summarized methods to avoid this complication to highlight the importance of not underestimating procedures labeled as “routine surgery” and to avoid unnecessary harm to patients.

## Case description

**Fig. 1:**
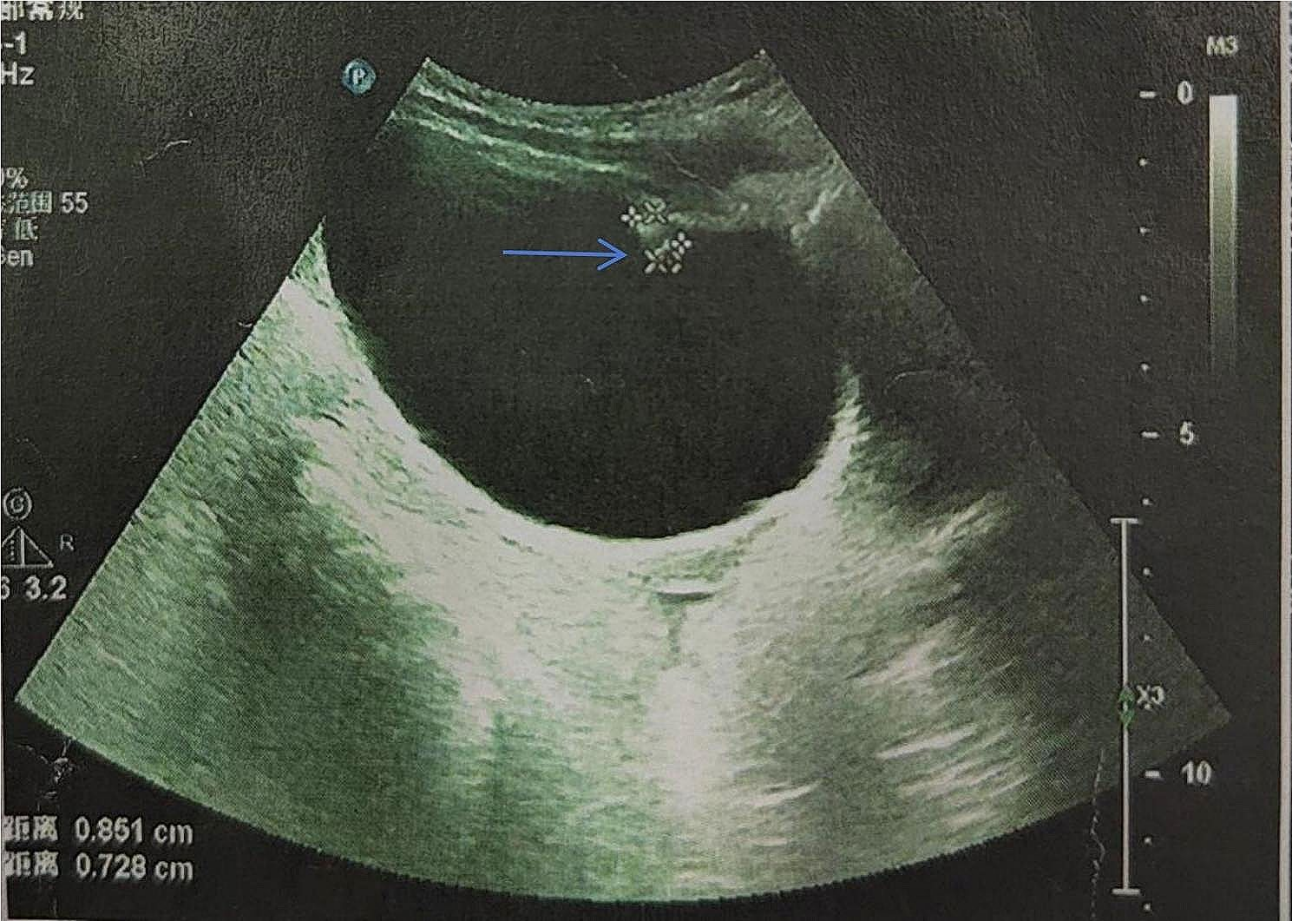
Left anterior wall of the bladder exhibited an uneven hyperechoic area (blue arrow), measuring about 8.5 mm*7.2 mm. CDFI depicted the absence of blood flow

A 4-year-old female child who experienced recurrent gross hematuria for over 2 months attended our outpatient department, accompanied by her mother. Inguinal hernia repair was performed at the local hospital two months ago following the incidental discovery of a reversible mass in the left groin area. Of note, an ultrasound examination was not performed prior to the surgical intervention. Notably, the patient developed gross hematuria two days post-surgery, characterized by the absence of polyuria, dysuria, urinary urgency, and abdominal pain. The patient was administered “cephalosporin” for 1 week, but hematuria persisted, with each episode lasting roughly 3–4 days. Nevertheless, hematuria was relieved by increased water intake or rest. A subsequent ultrasound examination performed at another hospital displayed uneven hyperechoic on the left anterior wall of the bladder, measuring approximately 8.5 mm*7.2 mm, with clear boundaries and homogeneous echoes. Meanwhile, CDFI revealed the absence of blood flow signals within the area, and a preliminary diagnosis of foreign bodies in the urinary bladder was made (see Fig. 1). Thus, the patient was admitted to our department two months after the initial surgery due to the presence of a foreign body in the bladder.

Physical examination exposed the presence of an oblique old surgical scar (about 2 cm) located near the symphysis pubis in the left lower abdomen. No swelling was observed in the left groin, and there were no expansile signs during crying. Abdominal examination showed no organ enlargement or abdominal fluid. Examination of the external genital examination yielded normal results.

**Fig. 2:**
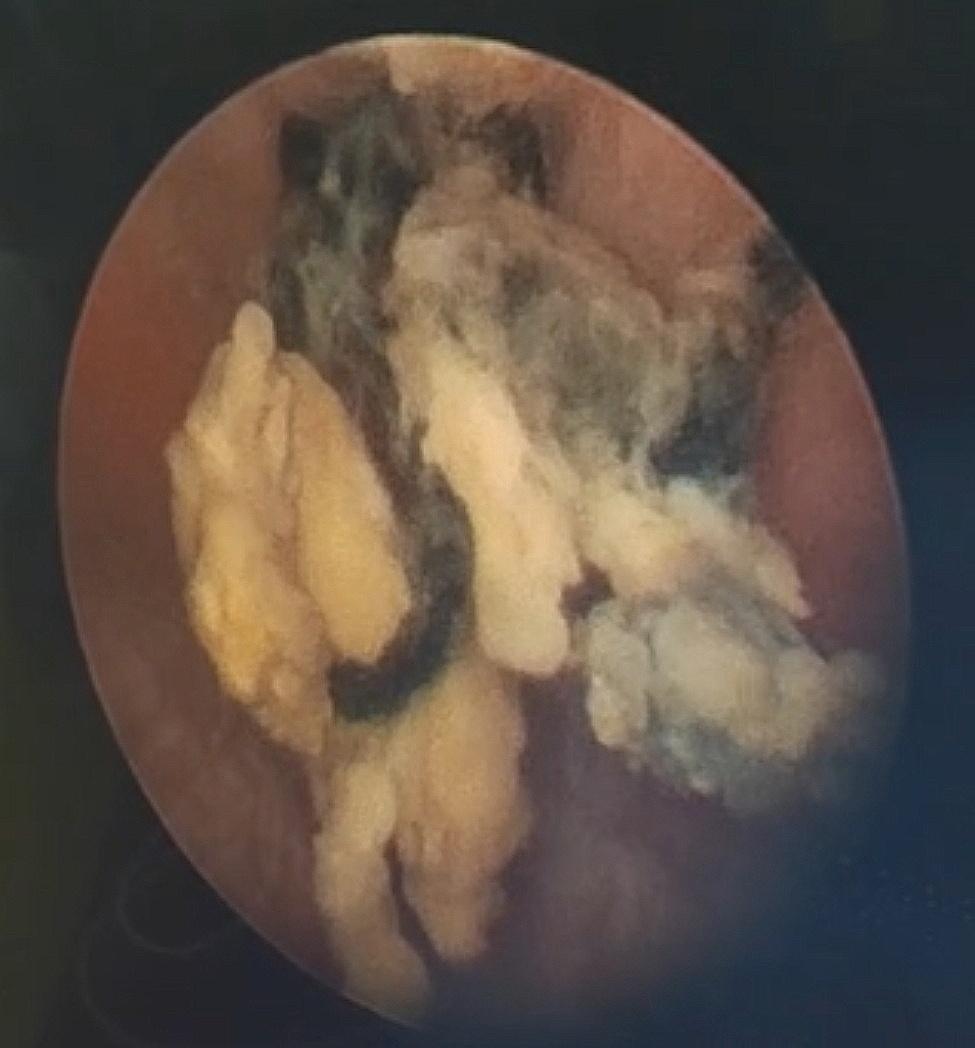
Two black suture knots, surrounded by calculi, were visualized in the left anterior wall of the bladder

**Fig. 3:**
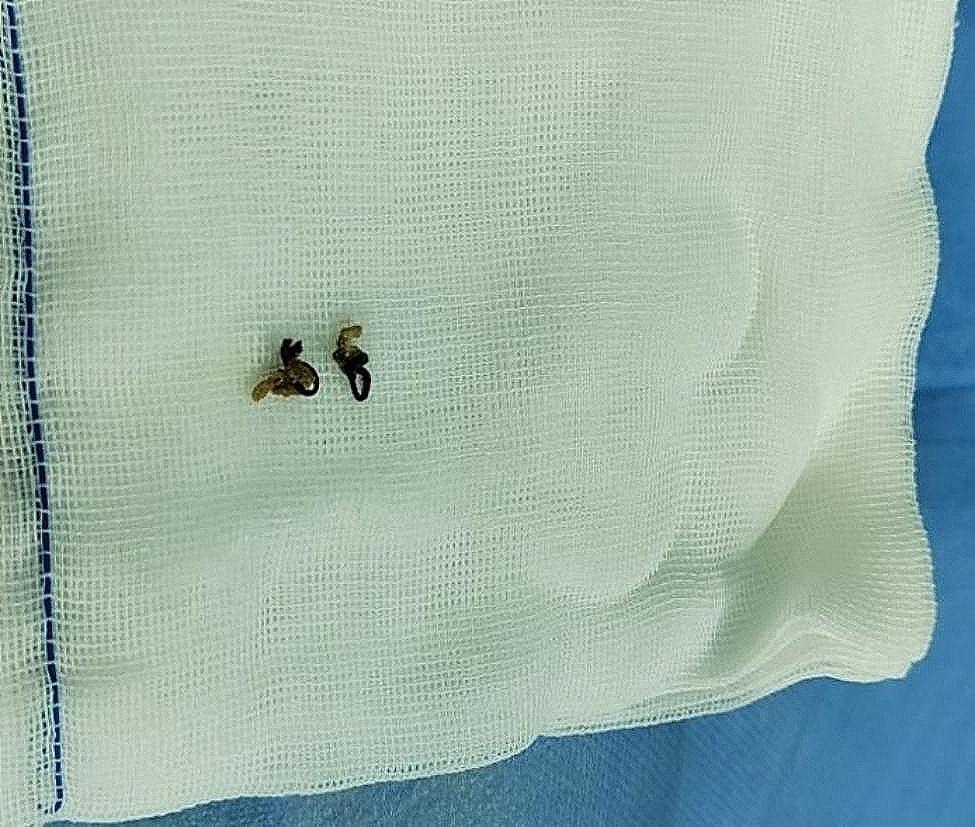
The knots and stones were successively extracted through the urethra

A cystoscopy examination was performed by a senior pediatric urologist in our hospital with 10 years of surgical experience. Briefly, a 10Fr cystoscope was inserted into the bladder through the external urethral opening. To avoid missing any foreign body on the bladder sidewall, physiological saline was slowly dripped to fill the bladder under direct vision. As anticipated, the mucous membrane in the triangle of the bladder appeared smooth, with the absence of stones, neoplasms, and diverticula. Moreover, the bilateral ureteral openings were normal. Upon further inspection, two black suture knots were noted in the left anterior wall of the bladder, surrounded by a few stones (Fig. 2). Besides, mild edema of the bladder mucosa was observed around the knots. The knots and stones were successively extracted using grasping forceps under the guidance of a cystoscope through the urethra (Fig. 3). During this procedure, it was noted that the bladder mucosa below the connection had healed (similar to the healing process observed in anal fistula suture cutting), with minimal bleeding and resistance encountered during the extraction. After the intervention, an 8Fr catheter was inserted. The surgical procedure lasted about 25 min. A second-generation cephalosporin was administered post-operatively to mitigate the risk of infection. The catheter was removed on the second day after surgery, and the patient was discharged on the 3rd day post-surgery.

The child was followed up for a month and did not experience gross hematuria. Ultrasonography revealed no abnormal echoes in the bladder. Urine analysis showed no red blood cells. In addition, no reversible mass was identified in the left groin area.

## Discussion

It is well-established that inguinal hernia repair is one of the most common surgical procedures in children [8]. Nevertheless, studies on bladder injury during inguinal hernia repair in the pediatric population are scarce. Sliding bladder hernias account for about 4% of inguinal hernias in adults [9] but are rare in children. According to an earlier study, 73% (22/30) of bladder injuries during inguinal hernia repair are diagnosed postoperatively [3], with roughly 73% (16/22) of these cases developing severe complications.

MUSTAFA et al. [10] reported that perforating sutures traversing the bladder wall or unrecognized bladder injury during dissection of the proximal end of the sac are the primary causes of paravesical abscess (PVA), a late complication of inguinal hernia repair. If not promptly diagnosed, they may evolve into potential infectious foci that promote the development of paravesical abscesses. Previous studies also hypothesized that the silk suture material employed for ligating the hernia sac facilitates PVA formation. Herein, the non-absorbable silk thread penetrated the bladder wall during inguinal hernia repair. Given that antibiotics were given following the onset of hematuria and that the sutures were completely removed 2 months after surgery, the patient did not develop PVA.

We were very fortunate that cystoscopy surgery was carried out within a specific time window following stone formation. Consequently, the stones were loose and small, contributing to easy removal. At the same time, due to a sufficiently prolonged time of knot cutting, no bleeding was observed in the bladder muscle and mucosal layer upon suture removal, and hematuria subsided the day following the surgery.

We postulate that bladder injury can be avoided by implementing the following measures: (1) Inguinal hernia repair is frequently regarded as a minor procedure and is usually delegated to resident or inexperienced surgeons [4]. However, even experienced surgeons can elicit bladder injury [11]. Therefore, surgeons should remain vigilant and focused to prevent technical errors that culminate in postoperative complications. (2) The surgeon needs to be familiar with the anatomical characteristics of the bladder in the pediatric population. In infants and young children, the bladder is positioned higher in the abdomen and descends to the level of the pelvis around the age of six. Furthermore, the bladder wall of children is thinner than that of adults, especially when the bladder is full. These anatomical features may lead to the misidentification of the bladder wall as a part of the hernia sac. Thus, emptying the bladder prior to surgery may reduce the likelihood of such misunderstandings. In addition, considering the proximity of the lateral side of the bladder to the internal inguinal ring, the incision site should be as lateral as possible. When ligating the hernia sac at a high level, particular attention should be paid to distinguishing the bladder wall. (3) Consider laparoscopic techniques: Since laparoscopy was first introduced to pediatric inguinal hernia repair in 1997, an increasing number of studies have validated its feasibility, and it has emerged as an effective approach for the treatment of inguinal hernias in children [12]. To the best of our knowledge, this is no report of bladder injury during laparoscopic hernia surgery in children. We believe that one of the benefits of laparoscopic hernia surgery is its ability to prevent bladder injury, as surgeons can visually confirm the position of the bladder during surgery. And if a sliding hernia is suspected before surgery, laparoscopic surgery should be the preferred choice.

## Conclusion

While the incidence of bladder injury during inguinal hernia repair in children is low, it can lead to severe complications. A comprehensive understanding of the unique anatomy of the inguinal region and the location of the bladder can assist in preventing accidental bladder injury.

## Data Availability

The details available from the corresponding author on reasonable request.
